# Reference assembly and gene expression analysis of *Apostichopus japonicus* larval development

**DOI:** 10.1038/s41598-018-37755-5

**Published:** 2019-02-04

**Authors:** Alexey V. Boyko, Alexander S. Girich, Marina G. Eliseikina, Sergey I. Maslennikov, Igor Yu. Dolmatov

**Affiliations:** 1 National Scientific Centre of Marine Biology, Far Eastern Branch, Russian Academy of Sciences, Palchevskogo 17, Vladivostok, 690041 Russia; 2 Far Eastern Federal University, Sukhanova 8, Vladivostok, 690950 Russia

## Abstract

The transcriptome of the holothurian *Apostichopus japonicus* was sequenced at four developmental stages—blastula, gastrula, auricularia, pentactula—on an Illumina sequencer. Based on our RNA-seq data and the paired-end reads from 16 libraries obtained by other researchers earlier, we have achieved the currently most complete transcriptome assembly for *A*. *japonicus* with the best basic statistical parameters. An analysis of the obtained transcriptome has revealed 174 differentially expressed transcription factors, as well as stage-specific transcription factors that are most promising for further study. In addition, a total of 1,174,999 high-quality single nucleotide polymorphisms have been identified, including 58,932 indels. A GO enrichment analysis of contigs containing polymorphic loci shows the predominance of GO terms associated with immune response. The data obtained by us provide an additional basis for a deeper study of the mechanisms of the planktotrophic-type development in holothurians and can be used in commercial sea cucumber breeding programs.

## Introduction

Echinoderms represent one of the ancient phyla of deuterostomes^1^. They manifest an ancient developmental pattern, through dipleurula-type larvae. In this connection, echinoderms are interesting model objects for studying this type of development. Among echinoderms, sea urchins and holothurians are of greatest interest as important objects of commercial fishing. For this reason, they are studied actively. The genome of the sea urchin *Strongylocentrotus purpuratus*^2,3^ is currently decoded and well characterized. There are numerous publications on the morphological and molecular features of development of *S*. *purpuratus* and other species of sea urchins.

Unlike sea urchins, development of holothurians has not been studied sufficiently. At present, the morphological features of larval development are described in detail for only two species, *Apostichopus (Stichopus) californicus* and *A*. *japonicus*^4–8^. There are works on expression of a number of genes in *A*. *japonicus* at different larval stages^9–11^. Recently, some works have been published on decoding of the genome of this species^12,13^; however, as the practice of eukaryotic genome sequencing shows, numerous additional researches are needed to obtain the final, tested and accurate genome assembly.

The holothurian *A*. *japonicus* is a valuable commercial species in China, ROK, DPRK, Russia, and Japan^14,15^. In addition to its value as a sea food product, this holothurian species is of interest as a source of biologically active substances^16^ and a model for the study of regeneration^13,17,18^. In spite of the large number of publications on this species, there are still very few works on its development. Apart from several studies on the morphology of larvae^4–7^, there are only two papers on the molecular mechanisms of development in *A*. *japonicus*^9,19^. Du *et al*.^19^ carried out transcrtiptome sequencing of samples representing different developmental stages and adult tissues; however, all the libraries were normalized and were not analyzed individually, which made it impossible to evaluate the expression of genes at different stages of development. A more detailed analysis of the development of *A*. *japonicus* was performed by Li *et al*.^9^. In the work, an attempt was made to identify differentially expressed genes (DEGs). Nevertheless, although individuals at all the developmental stages, from fertilized eggs to juveniles, were studied in this work, only one stage was actually analyzed, the transition from auricularia to doliolaria.

One of the fields of research implemented in aquaculture of these species is the search for molecular markers that can be useful in breeding works. Recently, single nucleotide polymorphisms (SNPs) have been considered as these markers^20^. In *A*. *japonicus*, a few hundred thousand to several million SNPs were identified, depending on the object of study, transcriptome or genome^12,13,19,21,22^. Nevertheless, these studies did not analyze the functions of genes with SNPs and the involvement of these genes in development.

In this work, we tried to obtain the most complete transcriptome, to determine DEGs at the main developmental stages of *A*. *japonicus* and the transcription factors (TFs) regulating it, as well as to identify SNPs and to establish the role of genes containing SNPs in development of this species.

## Results and Discussion

### De novo transcriptome assembly

Sequencing of four libraries, corresponding to the four developmental stages of the holothurian *A*. *japonicus*—blastula, gastrula, auricularia, and pentactula—resulted in a total of 230.8 million raw paired-end reads. After filtering and trimming adapters, 85% of paired-end reads were retained, with an average Phred quality score of 36. For further assembling, paired-end reads from 16 libraries from previous studies^12,21–24^ were added to these paired-end reads. As a result, the total number of raw paired-end reads was 629.2 million. Of them, 84% passed filtering and participated in assembling (see Supplementary Table S1). Unpaired reads that remained after filtering and read error correction, which amounted to 10% of the initial number of reads, were used in assembling, too. The average Phred quality score for all reads was 37; and average read length, 96 nucleotides.

**Figure 1:**
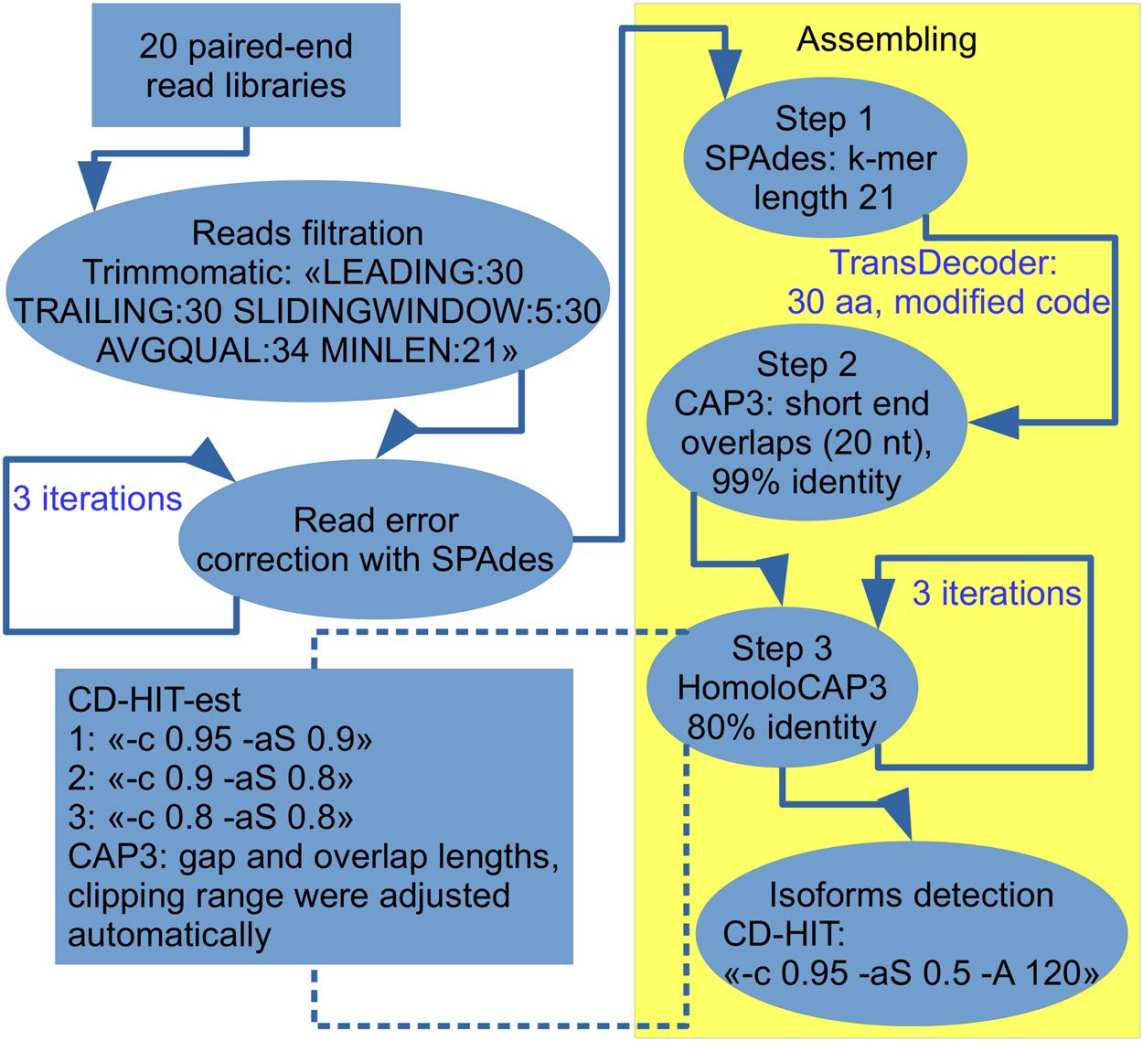
Scheme of the process of read filtering and assembling.

At the stage preceding the assembling, three iterations of read error correction were performed, which reduced the number of paired-end reads, however, with the loss percentage tending to zero (Fig. 1). The first stage of assembling in SPAdes resulted in a total of 703,169 contigs. This was unsatisfactory, as the level of fragmentation, the percentage of redundancy of almost identical contigs was high. Apparently, this situation arose due to the great number of polymorphisms, because we used reads obtained for different sea cucumber populations. Most of the variability came from 5′ and 3′ untranslated regions (UTRs), which interfered with the proper work of SPAdes and CAP3. For this reason it was decided to use only CDSs for further assembling. The entire area from the starting stop-codon to the terminal stop-codon, if any, was considered as a CDS. Only 371,845 contigs had CDS longer than 30 amino acid residues.

Also, all CDSs having hits of organisms other than echinoderms during BLAST search were removed. These contigs were to satisfy three conditions: the contig coverage should be above 70%; the identity to proteins of contaminant organism, above 80%; the lack of the best hits in available genomes of *A*. *japonicus*^12,13^.

**Figure 2:**
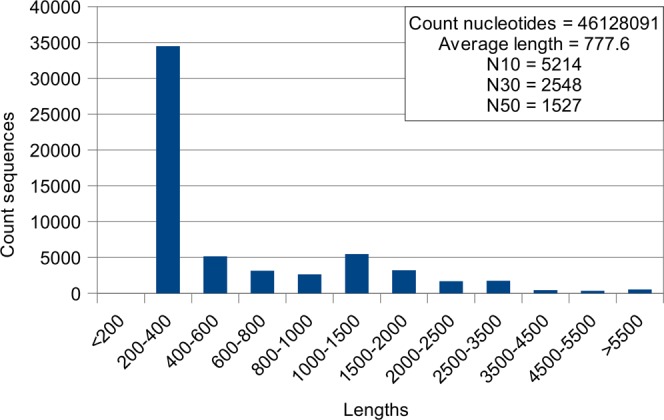
Length distribution of contigs and basic features of assembly.

All CDSs were then used to achieve the final assembly in HomoloCAP3. The script written by us is a software add-on to CAP3, which makes it possible to use the data of pre-clustering of sequences and automatically select some of CAP3 parameters. As a result of the work of HomoloCAP3, filtering of the contaminant sequences and subsequent clustering with the aim to identify isoforms, we obtained a total of 59,318 contigs and 53,267 genes (Fig. 2). HomoloCAP3 was found to be more efficient and fast than CAP3, because of pre-clustering of sequences, better use of the resources of modern computers, and automatic selection of CAP3 parameters such as overlap and gap lengths and clipping range. This approach to finalizing the assembly has been used for the first time and can significantly reduce the number of sequences with simultaneous increase in the number of full-length transcripts.

Since all the transcriptome assemblies have not only protein-coding sequences in their composition, only CDSs with a length of more than 200 nucleotides with significant BLAST hits in the sea urchin proteome were used in order to achieve a standardized comparison between assemblies. Our assembly includes, on average, about 95% of all protein-coding sequences of assemblies from previous studies (Du *et al*.^19^; Jo *et al*.^23^; Reich, Dunn, Akasaka, & Wessel, 2015; Z. C. Zhou *et al*.^22^, including genomic ones (Jo *et al*.^12^; X. Zhang *et al*.^13^. Compared to the above-listed assemblies, our assembly has better values of the basic statistic parameters such as average length, N50, N30, and N10 (Table 1). At the same time, all the previous assemblies cover an average of only 40% of our assembly, with the exception of the genome of *A*. *japonicus*^13^, for which this value was 70%. Hence, this assembly can serve as a reference assembly to search for polymorphisms, analyze gene families, etc. The standard statistical parameters of our assembly of *A*. *japonicus* transcriptome are also good, in spite of the dominant shift in the distribution of lengths towards the region of 200–400 nucleotides. Sequences of this range have almost no significant hits in searches against protein database, and probably represent a “trash” part of the assembly.

**Table 1:** Comparative analysis of *A. japonicus* assemblies.

Assembly	Year	Count CDSs	Count bases, 10^6^	Average length, nt	N10, nt	N30, nt	N50, nt	Coverage by our assembly, %	Coverage by assembly, %	Alignment reads, %
Our data	—	27598	35.2	1274	5865	3090	1946	100	100	28.6
NCBI ESTs	—	2816	1.6	569	1173	735	606	95	4	14
Du *et al*.^19^	2012	13724	10.7	776	1824	1224	912	97	22	16.2
Zhou *et al*.^22^	2014	26174	24.8	947	3513	1938	1317	98	46	25.8
Reich *et al*., 2015 (GAVS01.1)	2015	31611	29.2	922	3015	1743	1206	93	35	23.4
Jo *et al*.^23^ (HADD01.1)	2016	27670	27.4	991	3648	2097	1410	95	43	24.9
Jo *et al*.^23^ (HADE01.1)	2016	27445	23.5	856	2889	1725	1194	96	40	24.6
Jo *et al*.^23^ (HADF01.1)	2016	27396	23.4	855	3027	1746	1203	96	39	24.6
Jo *et al*.^12^ (genome)	2017	17111	18.1	1059	3531	1971	1380	91	35	17.7
Zhang *et al*.^13^ (genome)	2017	22643	29	1281	3891	2259	1551	88	70	21.4

### Differential expression analysis

**Figure 3:**
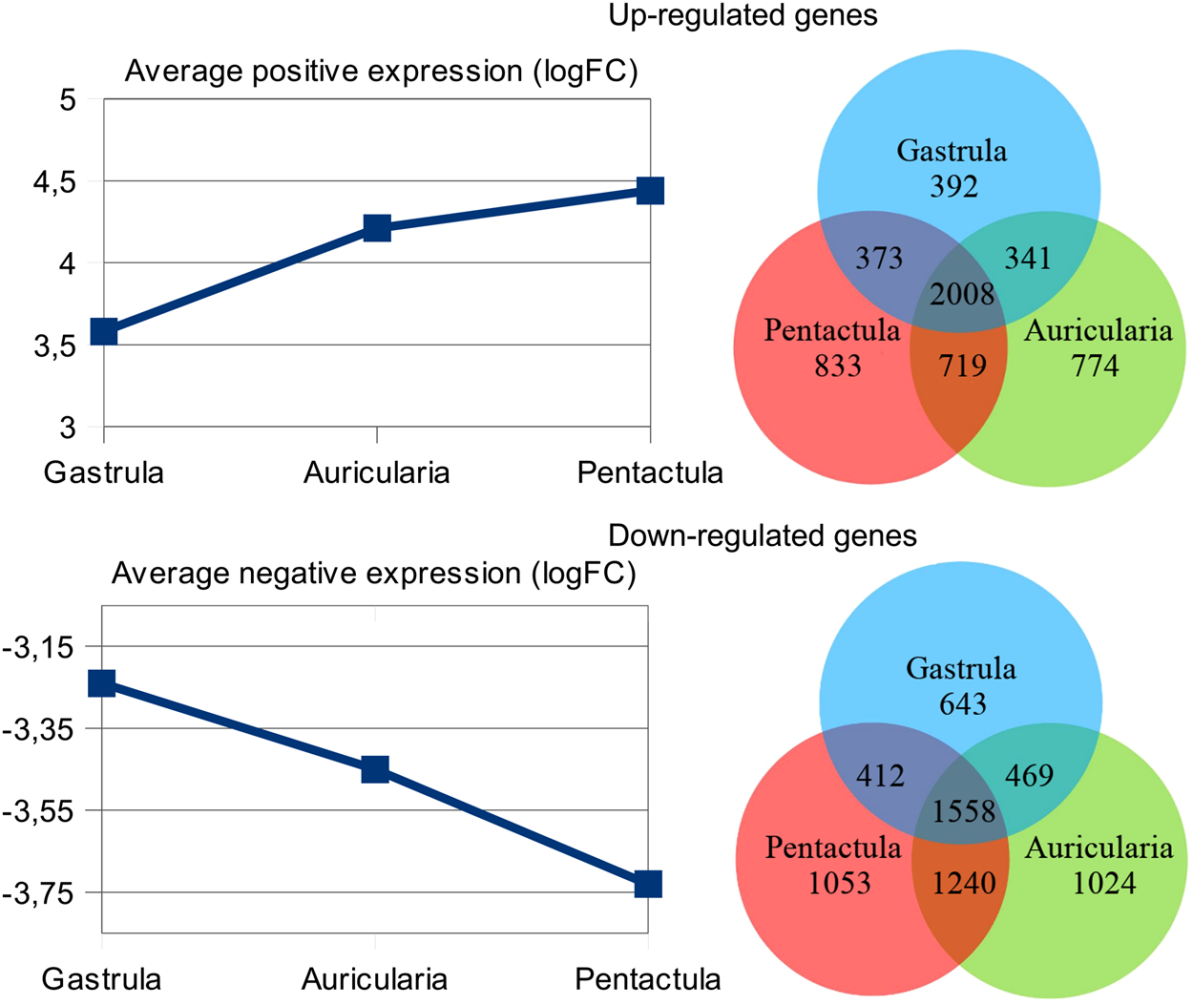
Average logFC values and Venn diagrams for up-regulated and down-regulated DEGs.

A search for DEGs revealed a total of 11697 genes with significant changes in expression level relative to the blastula stage (see Supplementary Table S2). Of these, 7867 sequences had significant hits against the NCBI NR database. The number of DEGs at the gastrula, auricularia, and pentactula stages is 6196, 8076, and 8196, respectively. The number of DEGs common to all three stages is 3566. The average value of logarithm of fold change (logFC) for both negatively and positively regulated genes grows insignificantly during the transition from blastula to pentactula (Fig. 3). This dynamics probably results from global changes in the work of genome of larval cells in the process of development, associated with the activation of zygotic genome, the gradual complication of the structure, and establishing of new tissues and organs. Obviously, the constant increase in unique DEGs in the sequence from blastula to pentactula can also be explained by the same processes.

### Annotation

Annotation of 59318 contigs by BLASTx searching against the NCBI protein non-redundant database resulted in identification of 29347 contigs and 25562 genes with significant hits, with 3541 contigs having only unnamed hits (see Supplementary Table S3). Hits belong to 2361 organisms; over a half of contigs matched echinoderm proteins (see Supplementary Table S4). In addition, there is a high probability of the presence of contaminant sequences. Even in the final version of the assembly, a number of sequences bear resemblance to proteins of Proteobacteria, Streptophyta, and Arthropoda (see Supplementary Table S4). However, the level of similarity and the lack of the verified and well-annotated genome in *A*. *japonicus* do not allow us to unambiguously define these contigs as a consequence of contamination.

Based on data of *A*. *japonicus* and *S*. *purpuratus* genomes^2,3,13^, it can be assumed that the number of *A*. *japonicus* genes varies from 22000 to 30000. The number of genes in our assembly can be estimated at 22000–25500, which fits into this framework. But this does not correspond to the data of Zhang *et al*.^13^, which suggest that *A*. *japonicus* has 30350 genes. The difference can be explained by the fact that, when compared with our assembly and with the *S*. *purpuratus* transcriptome, it turns out that some of the predicted genes in the work of Zhang *et al*.^13^ are probably not protein-coding ones. This is evidenced by the fact that out of the 30355 predicted genes, only 22643 genes have significant BLAST hits in the sea urchin transcriptome. In addition, both in our assembly and in the set of protein-coding genes obtained by Zhang *et al*.^13^, a part of the genes may be missing. In this regard, combining of our data with the data of Zhang *et al*.^13^ will provide the most complete understanding of *A*. *japonicus* genes.

**Figure 4:**
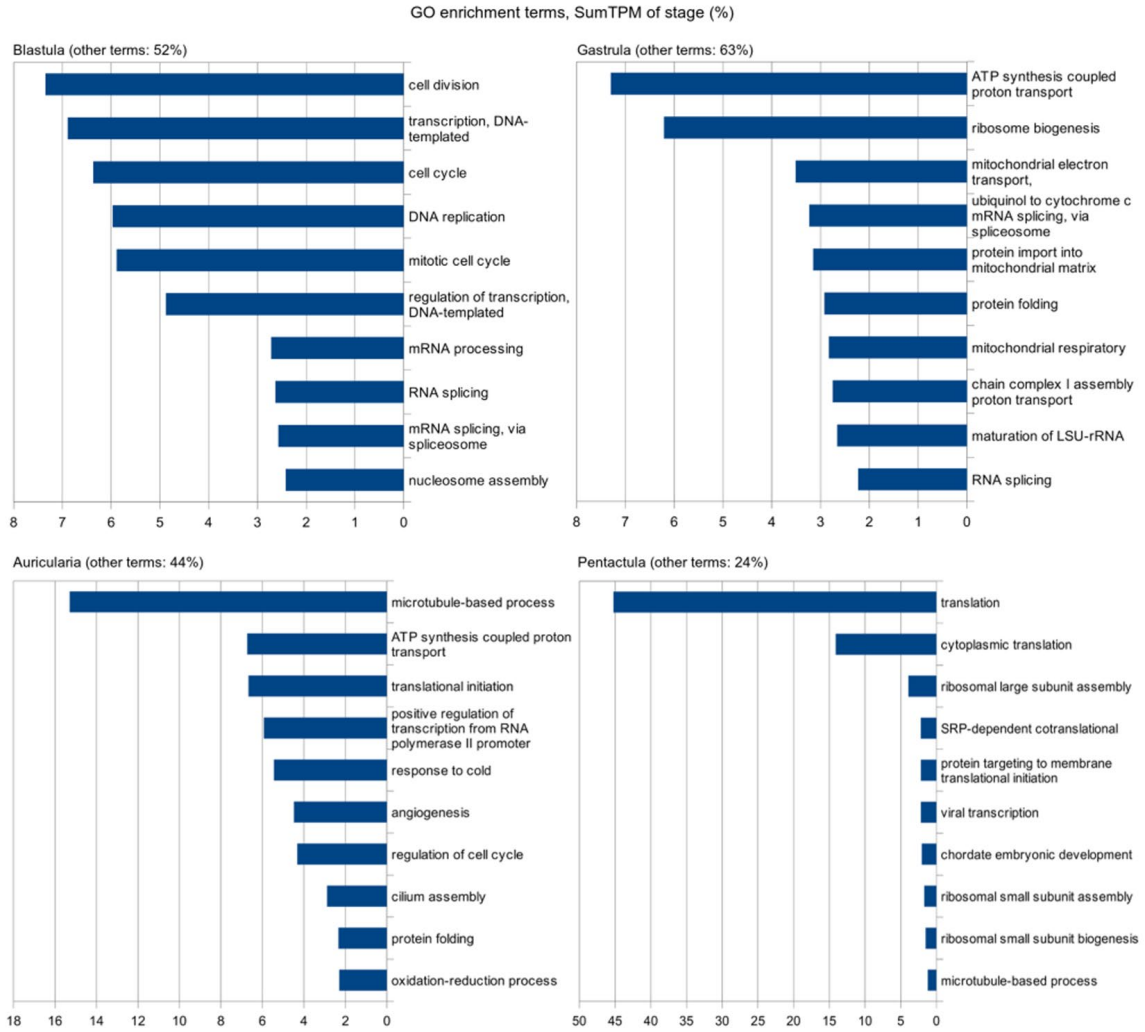
GO enrichment terms for four stages of development. The GO enrichment analysis for stages was modified to reveal the predominant, in terms of total TPM value, GO terms at a stage. Only the ten most predominant GO processes are shown.

During GO annotation, the number of contigs with hits insignificantly dropped as compared to BLASTx searching against the NCBI database. Of the 11697 contigs with significant changes in expression level, 6610 have hits in the SwissProt database and GO terms. The predominant terms at different stages of development of *A*. *japonicus* are shown at Fig. 4. Since the GO annotation is based on the functions of proteins of model organisms, GO enrichment analysis provides only a superficial understanding of the processes prevailing at one or another stage of development, the sites of their progress, and the functions in *A*. *japonicus*. For example, the GO-term “angiogenesis” is detected in the auricularia stage, although echinoderms lack blood vessels^25^. Nevertheless, this analysis is useful for an overall assessment of gene activity. Our modification of the standard approach to GO enrichment analysis made it possible to identify the most actively working stage-specific genes and to assume their function. Thus, the processes that correlate well with processes that actually occur at one or another developmental stage in holothurians are associated with a dozen of the dominant GO-terms. For example, most of the GO terms at the blastula stage are related to cell proliferation; at the pentactula stage, to translation.

### TFs searching and clustering of expression profiles

A search for homologues of sea urchin trascription factors resulted in identification of 293 TFs. Of them, 174 TF homologues have significant changes in expression level relative to blastula. All homologues were also verified against the NR NCBI database. Clustering of expression profiles resulted in 54 groups, many of which had already contained fewer than 100 sequences. In this regard, clusters having a similar expression pattern were manually combined. As a result, 19 clusters were obtained, each of which contained at least 200 sequences. Of these clusters, 13 included TFs; for this reason, only these ones were further analyzed.

**Figure 5:**
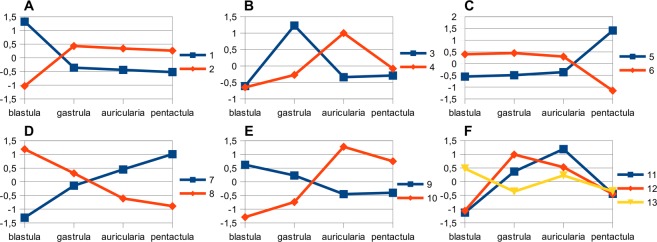
Expression profiles of gene clusters. (**A**) Clusters 1 and 2 having positive (cluster 1) and negative (cluster 2) peaks at blastula stage. (**B**) Clusters 3 and 4 having positive peaks at gastrula (cluster 3) and auricularia (cluster 4) stages. (**C**) Clusters 5 and 6 having positive (cluster 5) and negative (cluster 6) peaks at pentactula stage. (**D**) Clusters 7 and 8 with gradual increase and decrease in expression levels, respectively. (**E**) Clusters 9 and 10 having positive peaks at early (cluster 9) and later (cluster 10) stages of larval development. (**F**) Dynamics of expression of genes of clusters 11-13.

According to the dynamics of expression, clusters can be divided into three groups (Fig. 5). Clusters 1, 3–5 belong to the first group. They are characterized by one positive peak of expression at a certain stage of development. These clusters, apparently, most clearly show the features of the corresponding developmental stage, for this reason, only these ones were further analyzed. The second group includes clusters 7 and 8 with gradual increase and decrease in expression levels, respectively. Such a stable unidirectional variation in the activity of certain groups of genes obviously indicates global rearrangements in genome’s work that occur during the transition from embryo to pentactula. The third group was comprised of clusters with a more complex expression dynamics.

**Figure 6:**
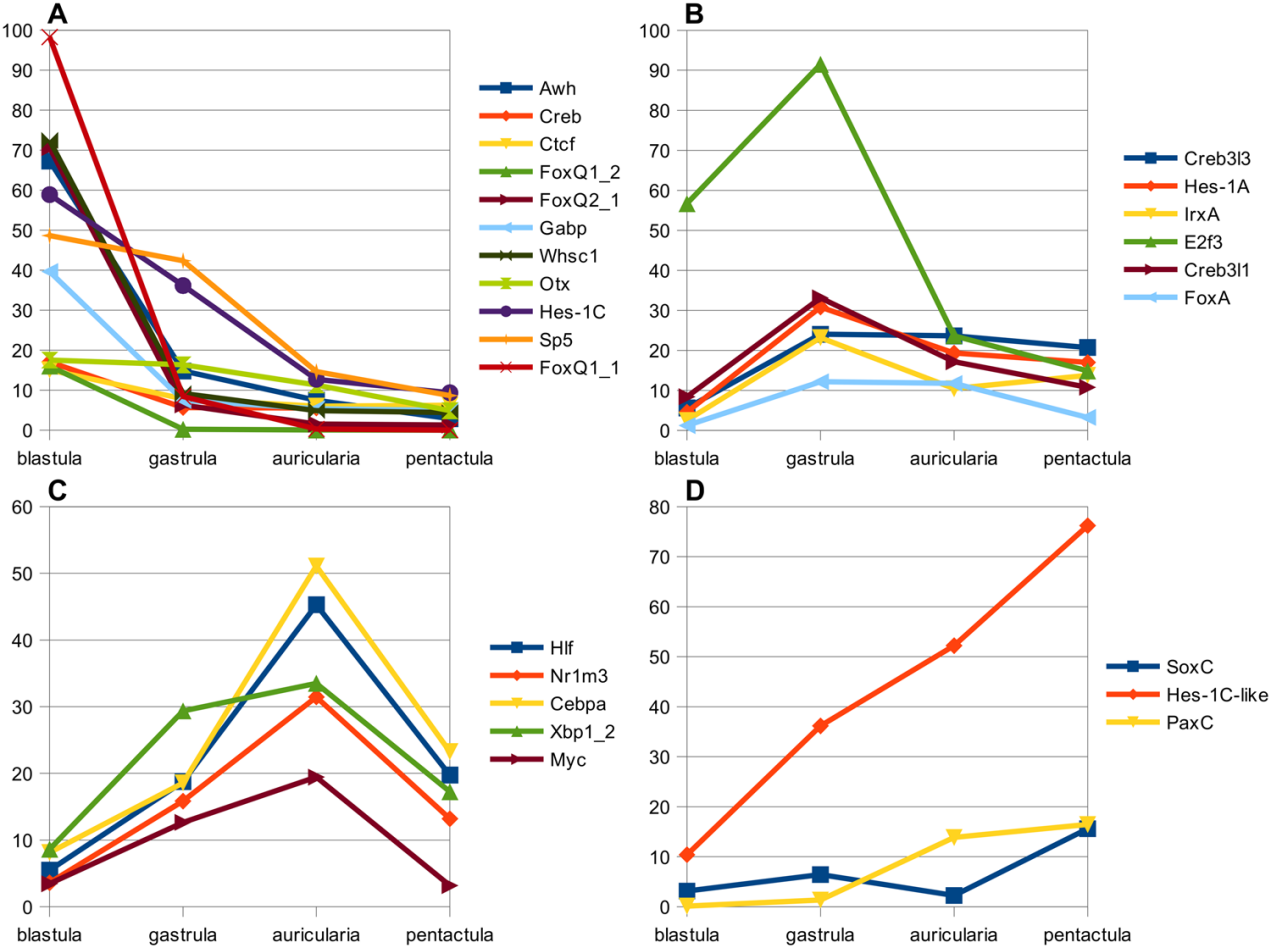
TPM values of 25 most predominant transcription factors. (**A**) TFs with peak of expression at blastula stage. (**B**) TFs with peak of expression at gastrula stage. (**C**) TFs with peak of expression at auricularia stage. (**D**) TFs with peak of expression at pentactula stage.

Moreover, as is indicated in Materials and Methods, threshold of TPM was used to identify the most vivid TFs in terms of expression. The expression profiles of 27 of these TFs with TPM values at each stage are shown in Fig. 6. The TPM threshold, used by us for TFs, on the one hand, focuses on TFs genes that are most actively expressed at one or another stage, and, on the other hand, artificially limits the diversity of TFs involved in development, which should be taken into account in further studies.

Apparently, the blastula stage in *A*. *japonicus* is largely characterized by cluster 1, which is formed by genes with maximum expression at this developmental stage (Fig. 5A). This cluster was the largest one by number of genes. It contained 2891 contigs and included a quarter of all detected DEGs. Since it is unknown when mid-blastula transition occurs in this species, the cluster can contain both maternal effect genes and genes of the embryo. However, the presence of such GO-terms as “regulation of transcription, DNA-templated” and “transcription, DNA-templated” in the cluster indicates activation of zygotic genes.

This cluster contained 37 TF genes, homologues of many of them play an important role in early development of sea urchins and holothurians^2,3,10,26–29^. Among these genes, genes of the *FoxQ* and *Hes1* families should be singled out (Fig. 6A). In *A*. *japonicus*, we found three transcripts that are close to FoxQ1 of *S*. *purpuratus*. They differ both in the amino acid sequence and in the intensity of expression. All the above facts allow us to assume the presence of three different *FoxQ1* genes in *A*. *japonicus* (we defined them as *FoxQ1_1*, *FoxQ1_2*, and *FoxQ1_3*), which probably emerged as a result of repeated duplication. This duplication supposedly reflects the differences in the functions of genes of the subfamily *FoxQ1* in holothurians.

As in the case of *FoxQ1*, we identified four contigs of Hes1, which have different degrees of homology to the three genes of the *Hes1* family in *S*. *purpuratus*: *Hes1A* (*Hairy2/4*), *Hes1B*, and *Hes1C*. According to the degree of homology to the *Hes1* gene family of *S*. *purpuratus*, we designated them as *Hes1A*, *Hes1A-like*, *Hes1C*, and *Hes1C-like*. The *Hes1A* and *Hes1C* genes probably have passed through a single duplication, which resulted in the presence of four *Hes1* genes in *A*. *japonicus*. At the same time, the fact that *A*. *japonicus* lacks the homologue of the third *Hes1* gene of *S*. *purpuratus* remains unclear. It is also worth noting that four *Hes1* genes, the best BLAST hits for which are Hes1C and Hes1A of sea urchin, have been found in the holothurian *Apostichopus* (*Parastichopus*) *parvimensis*, while the sea star *Patiria miniata* has only two *Hes1* genes with the same best hits^2,3^. Gene duplication and emergence of *Hes1B* in sea urchins might have occurred after the classes Echinoidea and Holothuroidea diverged. It is an interesting fact that the *Hes1C* and *Hes1C-like* genes in *A*. *japonicus* have opposite expression profiles in development (Fig. 6). Thus, a detailed clarification of the functions of *FoxQ* and *Hes1* families in the development and evolution of echinoderms can bring interesting results.

For cluster 3, an increase in expression was recorded at the gastrula stage (Fig. 5B). GO enrichment analysis revealed only two terms: “response to drug” and “DNA integration”. The cluster has a total of 312 sequences and 1 TF (Nkx3-2), the expression of which was too low to pass filtering for minimum TPM value.

The genes forming cluster 3 and having a specific surge of expression at the gastrula stage are not capable of causing large-scale rearrangements typical of this stage. Apparently, this cluster should be regarded as an indicator of the processes prevailing in this developmental period. The GO term “Response to drug”, probably, indicates larva’s competence to receive and/or respond to external effects, since the GO term “response to chemical” is a parental term for “response to drug”. In *A*. *japonicus*, the embryonic period ends at the late blastula stage, when larva hatches from the eggshell^7^. Accordingly, larvae enter the environment and must respond to increased external impact.

The GO term “DNA integration” probably indicates a greater activity of transposons in gastrula than in the other larval stages of *A*. *japonicus*. It is known that transposons have an effect on the activity of some genes, pluripotency and cell fate, and participate in regeneration and asexual reproduction in holothurians^30–32^.

Most of the genes involved in gastrulation and other processes occurring during this period are highly active at other stages also; as a result, their expression profiles become “blurred” and isolating the “gastrula-specific” genes is quite difficult. This apparently is the cause of the presence of only one TF in the cluster with a specific surge of expression at the gastrula stage, Nkx3-2, whose expression level is low compared to the rest of the TFs involved in the development. In this case, some TFs found in other clusters have the highest expression values at this stage (Fig. 6B) and, obviously, participate in regulation of the formation and functions of gastrula in *A*. *japonicus*. Our results on the expression of genes *IrxA*, *Hes1A*, *E2f3*, *FoxA*, *Creb3l3*, and *Creb3l1* are consistent with the results obtained for sea urchins and holothurians^29,33–37^.

The auricularia stage is the longest in the larval development of holothurians. During this period, the larval digestive system is formed, the shape and dimensions of the larva become more complicated, its ciliary bands elongate, and hyaline spheres develop. In late auricularia, active development of mesodermal structures begins. The growth of the auricularia occurs through increase in the amount of extracellular matrix in the primary body cavity.

At the auricularia stage, genes of cluster 4 show positive peak in expression (Fig. 5C). This cluster contained 1318 contigs and 24 TFs. The vast majority of GO terms are associated with various aspects of fat metabolism and transport of various substances. In general, the TPM value for genes of this cluster was lower than in other clusters at all the stages, which is also true for the TFs genes of this cluster. Thus, none of the TFs passed filtering for minimum TPM value. Nevertheless, as in the case of gastrula, other clusters have TFs with high expression level at the auricular stage: *Hlf*, *Nr1m3*, *Myc*, *Cebpa*, and *Xbp1_2* (Fig. 6C). Almost all of these genes, except for *Xbp1*, are involved in the regulation of development in sea urchins^33,38–41^. In *A*. *japonicus*, a large number of *Xbp1_2* transcripts are found not only in auricularia, but also in blastula and pentactula, which indicates its involvement in regulation of morphogenesis.

At the pentactula stage, larvae settle and pass over to the benthic mode of life. The definitive pattern of the body has already formed, but the processes of morphogenesis and growth of organs and the differentiations of the constituent cells are active^4,5^. The highest gene expression at the pentactula stage was observed for cluster 5 (Fig. 5C). This cluster included 972 contigs and 16 TFs. These genes are involved in such processes as translation, angiogenesis, cell adhesion, positive regulation of cell migration, methylation, organ development, and regulation of some signaling pathways. Expression of these genes is dozens and hundreds times as high as that at all the other stages. Significant increase in activity of the DNA methylation processes is apparently connected with the final states of cell differentiation. Among TFs of this cluster, the highest expression is shown by SoxC (Fig. 6D). In addition, among the TFs of the other clusters, the highest value of TPM at the pentactula stage was recorded for PaxC и Hes1С-like. SoxC and PaxC in the sea urchin participates in the specification of neural precursor cells and skeletal morphogenesis^42,43^.

### SNP detection

A search for SNPs revealed a total of 1,174,999 high-quality polymorphisms, including 58932 indels (Table 2). When SNP was detected in *A*. *japonicus*, it turned out that the number of polymorphisms, estimated for all the populations together, was significantly larger than the sum of polymorphisms of all the populations. This can be explained by the fact that, when working with individual populations, filtering for coverage of each SNP cuts most of them. In this regard, drawing a conclusion about the genetic proximity of populations is unjustified if based on these values. The transition/transversion ratio for all data is 1.5; for some populations, about 1.8, which is higher than that mentioned in previous studies^19,21,22^. The frequency of polymorphisms was 33.57 per kilobase. This is two times as high as that in previous studies of transcripts^19,22^ and one and a half times as high as that in genomic studies^12^. This discrepancy is explainable when taking into account the fact that our assembly was performed using data from different sea cucumber populations, whereas the previous studies were performed based on only one population. All values were only slightly lower when polymorphisms were counted in contigs with significant BLAST hits against the NR NCBI protein database.

**Table 2:** SNPs of different populations of *A. japonicus*.

Population	All contigs				Contigs with hits			
	Count SNPs per kb	Transition	Transversion	Indel	Count SNPs per kb	Transition	Transversion	Indel
Russian	5.04	77164	41755	3874	4.93	71309	36840	2007
Chinese	6	117064	63519	5144	6	107463	55873	2976
South Korean	5.85	101518	57111	5996	5.76	92103	49158	3417
All	33.57	689365	455283	58932	32.16	605735	379302	36931

**Figure 7:**
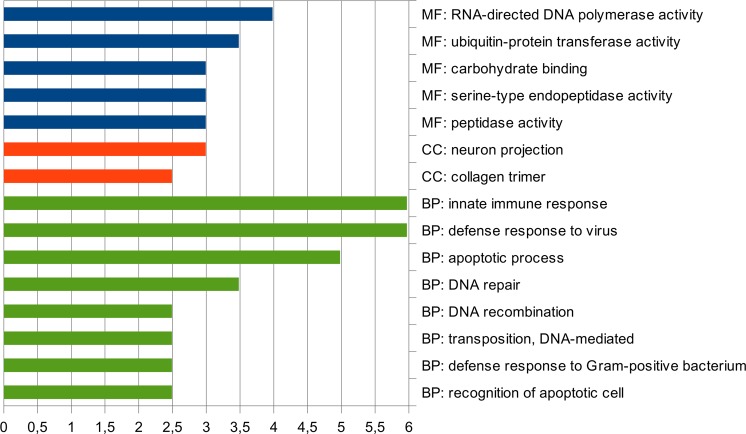
GO enrichment terms for highly polomorphic genes. On the abscissa axis is the percentage of the total number of contigs of the test set.

Of the 9334 differentially expressing genes with polymorphisms, 6610 had significant hits against the SwissProt database. Among these genes, only 201 contigs had a frequency of polymorphisms two times as high as the average. GO enrichment analysis, performed for these contigs, showed the predominance of terms related to immunity, DNA repair, and apoptotic processes (Fig. 7). This makes it possible to widely use genetic markers of health for industrial cultivation of this species and requires more detailed research. Small number of highly polymorphic genes indicates a high selection pressure on genes important for development and can much simplify creation of genetic markers of health.

## Methods

### Animals

Adult mature individuals of the holothurian *Apostichopus japonicus* (Selenka, 1867) were collected in Peter the Great Bay, Sea of Japan, and kept in 3 m^3^ tanks with running aerated seawater at 16 °C. Spawning was induced by thermal stimulation^15^. Zygotes were transferred to 370-liter tanks with aerated seawater at 20–22 °C, where they developed. Larvae were fed with the microalga *Dunaliella salina*.

### Sample collection and RNA extraction

For the analysis, larvae at the following stages were used: blastula, gastrula, auricularia, and pentactula. A total of about 100 larvae from each of the stages were selected. Fixation was performed in 3 mL of RNA Later (Sigma, USA) for 24 h at 4 °C; then the material was stored at –20 °C. Before isolating total RNA, the larvae were precipitated in sterile seawater. Homogenization was carried out with metal balls on a TissueLyser LT homogenizer (Quagen, Germany). Total RNA was isolated using phenol-chloroform extraction^44^.

### Transcriptome sequencing

After testing the total RNA on an Agilent TapeStation (Agilent, USA), the libraries were prepared using a TruSeq Stranded mRNA Library Prep Kit (Illumina, USA), and fragments with a length of 200–450 nucleotides, including adapters, were selected. After testing the quality on an Agilent TapeStation, paired-end sequencing (2 × 100) was performed on an Illumina HiSeq2500. Raw reads were uploaded to the NCBI SRA database under the accession numbers SRR6075437, SRR6075438, SRR6075435, and SRR6075436 for the blastula, gastrula, auricularia, and pentactula stages, respectively.

### De novo transcriptome assembly

In order to obtain the most complete and accurate transcriptome assembly, in addition to the four libraries of paired-end reads, obtained by us, we used also the currently available data, including 7903 ESTs from the NCBI database, contigs assembled from 454 reads^19^, and 16 libraries of raw illumina paired-end reads (listed in Supplementary Table S1) from previous studies^12,21–24^. Raw reads in FASTQ format were processed using Trimmomatic 0.36^45^ with “LEADING:30 TRAILING:30 SLIDINGWINDOW:5:30 AVGQUAL:34 MINLEN:21” parameters to obtain clean reads by removing those containing adapter sequences, poly-N sequences, or low-quality bases.

These high-quality reads were de novo assembled using SPAdes 3.10.1^46^ with 3 iterations for read correction step and with a k-mer length of 21. Of all the obtained contigs, as well as ESTs from NCBI and contigs from the study of Zhou *et al*.^22^, the coding sequences (CDSs) with a minimum length of 30 amino acid residues were extracted using TransDecoder 4.1.0 (http://transdecoder.github.io/). The code of TransDecoder was modified in such a way that stop-codon at the beginning of sequence but not “ATG” (Met) mark is an indicator of the beginning of CDS. All CDSs were verified using BLAST-search^47^ against the Uniref90 database, as is described in the manual to TransDecoder.

Then all the obtained sequences were subjected to the assembling stage in CAP3^48^ for finding short end overlaps (>20 nt) with a 99% identity. Subsequently, the obtained sequences were clustered into CD-HIT 4.7^49,50^ with three iterations: at the first iteration, sequences were clustered into CD-HIT with the parameters “-c 0.95 -aS 0.9”; at the second, “-c 0.90 -aS 0.80”; at the third, “-c 0.80 -aS 0.80”. After each iteration, sequences in the clusters were assembled with an identity threshold of 80%, using the own Python script, defined by us as HomoloCAP3. This script is a software add-on to CAP3 that makes it possible to use the data of pre-clustering of sequences and automatically select CAP3 parameters such as overlap and gap lengths and clipping range. After the final iteration, assembled CDSs were again clustered into CD-HIT with the parameters “-c 0.95 -aS 0.5 -A 120” for revealing the transcript isoforms. The resulting sequences were filtered according to the NCBI requirements and uploaded to the NCBI TSA database with the index GFXQ00000000.1. To compare assemblies, we used only CDSs with a length of over 200 nucleotides having significant BLAST hits in the sea urchin proteome.

### Differential expression analysis

To find DEGs, a standard pipeline from the Trinity v2.4.0 software^51^ was used; the number of mapped reads was calculated in RSEM v1.3.0^52^; paired-end reads aligning was performed in Bowtie 2.2.9^53^; the following parameters were added to the default ones: “-L 25 -N 1 –minins 50 –maxins 600”. A Trinity’s standard procedure for detection of significant changes in expression was modified. Thus, only sequences with more than 20 mapped paired-end reads were included in the analysis; also, the number of mapped reads would be enough for a 10-fold coverage of each nucleotide of sequence, with a zero number of mapped reads allowed at any stage. After this filtration, differential expression was evaluated for the sequences in edgeR 3.6^54^ with a specified level of dispersion of 0.1; those DEGs, the expression level of which was two times as high at each of the stages as that at the blastula stage, and with a Padj value lower than 0.05, were considered actual.

### Annotation

Annotation was carried out against several protein databases with a standard e-value of 1e-6 for BLASTX 2.2.30. Basic annotation was performed against the NCBI NR database (19.09.17); GO annotation was carried out against the SwissProt database (20.10.17); the annotation for finding the transcription factors and comparative analysis of assemblies was based on sea urchin proteins from the EchinoBase project^2,3^. To identify the most vivid TFs in terms of expression, only the TFs with values of TPMs (Transcripts Per Kilobase Million) more than three times as high as the mean TPM per stage among all the TFs, were taken for consideration.

GO enrichment analysis was carried out in GOAtools 0.6.10^55^ with the key “–no_propagate_counts”; then its output was filtered for the number of sequences with this GO term (fewer than 5) and for the ratio of the percentage of GO term in test to the percentage in reference (lower than 1.5). The reference set was all DEGs.

For developmental stages, the GO enrichment analysis was modified to reveal the predominant, in terms of total TPM, GO terms at a stage. For this, only the DEGs with TPM higher than the mean value at all stages and higher than unity were selected for each of the stages. The list of these sequences for a stage formed a test set. Then the output of goatools was filtered according to the same criterion, but with separate GO terms used instead of sequences.

In the case of cluster analysis, the test set was the cluster. In the case of analysis of SNPs, the test set was contigs with a polymorphism frequency of 2 times as high as the mean.

### Clustering by expression profiles

For clustering, only transcripts with significant variations in expression were used. The clustering was carried out with an 80% threshold of profile matching, using scripts from the Trinity package. Then the clusters similar in profile were manually combined into a single one. Eventually, only the clusters with at least one TF in them, as well as with more than 20 sequences annotated in the GO database were retained.

### Search for polymorphisms

The search for polymorphisms was carried out separately for each of the three populations: Russian, Chinese and South Korean, as well as for all of them together (see Supplementary Table S1). Paired-end reads aligning was performed in BWA 0.7.17^56^ with the parameters “-k 21 -c 50 –M”; after that, only the paired aligned reads with an alignment quality (MAPQ) higher than 10 were selected using SAMtools 1.3.1^57^. Then aligned reads were processed using Picard 2.15^58,59^ to remove all duplicates. Search and validation of SNPs were performed in GATK HaplotypeCaller v3.8^58,59^, according to the protocol recommended by the developers for RNA-seq data. Besides the recommended filters, additional ones with different coverage (DP < 20) and quality of alignment (MQ < 40) were introduced. The further analysis was performed using VCFtools v0.1.13^60^ and Python 3.4.

## Supplementary information

Supplementary Information

<b>Table S1</b>

<b>Table S2</b>

<b>Table S3</b>

<b>Table S4</b>

## Data Availability

The raw reads, obtained by us, were uploaded to the NCBI SRA database under the accession numbers SRR6075437, SRR6075438, SRR6075435, and SRR6075436 for the blastula, gastrula, auricularia, and pentactula stages, respectively. The assembly was uploaded to the NCBI TSA database under the accession number GFXQ00000000.1. The additional paired-end reads, used in the assembling, are listed in Supplementary Table S1.
