# Supplementary material for: Reference assembly and gene expression analysis of Apostichopus japonicus larval development
Source: Sci Rep. 2019 Feb 4;9:1131. doi: 10.1038/s41598-018-37755-5 (PMC6362246; doi:10.1038/s41598-018-37755-5)
Supplement: Supplementary file 1 — Supplementary Information [file 41598_2018_37755_MOESM1_ESM.pdf]

Reference assembly and gene expression analysis of Apostichopus japonicus larval development.

Alexey V. Boyko[1,2,*], Alexander S. Girich[1,2], Marina G. Eliseikina[1], Sergey I. Maslennikov[1,2] , Igor Yu. Dolmatov[1,2]

Table S1. The basic features of analyzed Illumina paired-end libraries before and after filtration

Population	Study	SRA index	Raw reads			Filtered reads		
			CountReads, 10^6	AvgLength	AvgPhredScore	CountReads, 10^6	AvgLength	AvgPhredScore
Russian	Our data	SRR6075437	55,6	100	35,4	47,2	97,7	35,9
		SRR6075438	62,8	100	35,4	53,7	97,4	35,9
		SRR6075435	52,5	100	35,4	45	98,1	35,9
		SRR6075436	59,9	100	35,4	50,3	97,6	35,9
Korean	Jo et al., 2016	ERR1193930	32,8	101	36,1	29,3	95	37,5
		ERR1193931	29,5	101	36,1	26,3	95	37,5
		ERR1193932	28,4	101	36,1	25,3	94,9	37,5
	Jo et al., 2017	SRR2442873	29,5	101	36,1	26,4	95	37,5
		SRR2442874	32,8	101	36,1	29,3	95	37,5
		SRR2442875	28,4	101	36,1	25,3	94,9	37,5
Chinese	-	SRR1185973	6,7	90	33,1	5,6	78	36
	-	SRR1216681	28,3	76	26,4	15,3	66	37,1
	Zhou et al., 2016a	SRR2089767	36,4	126	34,8	27	117,7	36,7
		SRR2128002	30,7	126	35	25,9	113,1	36,5
	Zhou et al., 2016b	SRR2128001	34	126	34,9	28,6	112,6	36,4
	Zhou et al., 2014	SRR414926	13,4	90	36	11,6	82,3	37,4
		SRR414927	13,4	90	36,1	11,7	82,6	37,4
		SRR414928	13,4	90	36	11,6	82,4	37,4
		SRR414929	13,8	90	36,1	11,9	82,2	37,4
		SRR414930	26,9	90	35,6	23	79,8	37,3

Table S2. Number of mapped reads per contigs and developmental stages

Table S3. Results of BLASTx searching of best hits against NCBI protein non-redundant database.

Table S4. Taxonomic distribution of hits in Protein non-redundant NCBI database

**Phylum**	**Count sequences**	**Percent identity**	**Sequences coverage**	**Class**	**Count sequences**	**Percent identity**	**Sequences coverage**
Echinodermata	15519	53.11	78.71	Asteroidea	7821	52.69	79.39
Chordata	3833	53.49	75.94	Echinoidea	6770	51.74	77.86
Proteobacteria	1734	66.75	89.49	Actinopteri	1796	58.42	77.06
Arthropoda	1248	55.39	77.79	Anthozoa	1121	47.46	77.95
Cnidaria	1176	47.46	78.04	Gammaproteobacteria	1067	66.36	90.35
Streptophyta	1173	81.46	88.32	Enteropneusta	926	49.99	76.71
Hemichordata	926	49.99	76.71	Holothuroidea	917	66.81	79.28
Mollusca	877	49.12	75.85	Insecta	646	53.86	75.89
Brachiopoda	343	49.17	75.36	Alphaproteobacteria	543	68.3	89.13
**Superkingdom**	**Count sequences**	**Percent identity**	**Sequences coverage**	Bivalvia	542	48.54	77.43
Eukaryota	27036	54.51	78.93	Mammalia	495	52.09	73.96
Bacteria	2186	65.31	87.64	Lingulata	342	49.06	75.42
				Aves	258	52.52	76.83
				Gastropoda	257	49.23	73.38
